# Lexical effects on talker discrimination in adult cochlear implant usersa)

**DOI:** 10.1121/10.0025011

**Published:** 2024-03-01

**Authors:** Terrin N. Tamati, Almut Jebens, Deniz Başkent

**Affiliations:** 1 Department of Otolaryngology, Vanderbilt University Medical Center, 1215 21st Ave S, Nashville, Tennessee 37232, USA; 2 Department of Otorhinolaryngology/Head and Neck Surgery, University Medical Center Groningen, University of Groningen, Groningen, The Netherlands; 3 Research School of Behavioral and Cognitive Neurosciences, Graduate School of Medical Sciences, University of Groningen, Groningen, The Netherlands

## Abstract

The lexical and phonological content of an utterance impacts the processing of talker-specific details in normal-hearing (NH) listeners. Adult cochlear implant (CI) users demonstrate difficulties in talker discrimination, particularly for same-gender talker pairs, which may alter the reliance on lexical information in talker discrimination. The current study examined the effect of lexical content on talker discrimination in 24 adult CI users. In a remote AX talker discrimination task, word pairs–produced either by the same talker (ST) or different talkers with the same (DT-SG) or mixed genders (DT-MG)–were either lexically easy (high frequency, low neighborhood density) or lexically hard (low frequency, high neighborhood density). The task was completed in quiet and multi-talker babble (MTB). Results showed an effect of lexical difficulty on talker discrimination, for same-gender talker pairs in both quiet and MTB. CI users showed greater sensitivity in quiet as well as less response bias in both quiet and MTB for lexically easy words compared to lexically hard words. These results suggest that CI users make use of lexical content in same-gender talker discrimination, providing evidence for the contribution of linguistic information to the processing of degraded talker information by adult CI users.

## I. INTRODUCTION

Real-world speech communication involves interacting with talkers with diverse voices and accents, often in the presence of environmental noise or competing talkers (e.g., Mattys *et al.*, 2012). Indexical information encoded in the speech signal conveys information about talker traits, including a talker's identity, emotional state, and social status (Abercrombie, 1967; Bent and Holt, 2017). Sensitivity to indexical information in speech plays an important role in speech perception and spoken word recognition. Normal-hearing (NH) listeners make use of both linguistic information and indexical information to uncover the intended meaning of an utterance (Nygaard *et al.*, 1994; Nygaard and Pisoni, 1998) as well as to make judgements about a talker's identity and the talker's physical, emotional, and social traits (e.g., Labov, 1973; Lass *et al.*, 1976; Ptacek and Sander, 1966; Van Lancker *et al.*, 1985a; Van Lancker *et al.*, 1985b). For hearing-impaired adults with cochlear implants (CIs), the paucity of indexical cues transmitted by their devices may alter how they process indexical information as well as their reliance on linguistic information in indexical processing (Gaudrain and Baskent, 2018), particularly in noisy environments (Cullington and Zeng, 2008; El Boghdady *et al.*, 2019). The current study examined talker discrimination in adult CI users and the impact of lexical information on talker discrimination in quiet and in MTB.

Several lexical and phonological factors, such as lexicality or word status (i.e., real word vs nonword) or phonotactic probability, have been shown to impact the processing of talker-specific details that NH listeners use for discriminating or identifying talkers. NH listeners are more sensitive to talker-specific details in talker discrimination for real words compared to nonwords (El Adas and Levi, 2022). Talker discrimination judgements are also faster for words with a high phonotactic probability, compared to words with low phonotactic probability and nonwords (El Adas and Levi, 2022). Further, previous studies demonstrating differences in voice or talker discrimination for time-reversed speech, compared to forward speech (Jebens *et al.*, 2022; Koelewijn *et al.*, 2021; Perrachione *et al.*, 2019), suggest that phonological processing impacts talker discrimination, since the time-reversal affects different phonetic features at the segmental and suprasegmental level.

Similarly, lexical and phonological effects can be observed across other indexical processing tasks, to a varying degree. Word status has been shown to alter talker identification in a voice line-up task including a talker training (El Adas and Levi, 2022). However, word status was not found to alter voice cue weightings in a perceived voice gender categorization task (Jebens *et al.*, 2022). Other task manipulations, such as introducing variability in stimulus materials (vs fixed stimulus materials), have been shown to impact talker (Cleary and Pisoni, 2002) and voice cue discrimination (Koelewijn *et al.*, 2021; Zaltz, 2023), further suggesting that the linguistic and/or acoustic content of the utterances impacts talker and voice cue perception. Finally, Levi and colleagues (2007) found that the lexical difficulty of the target word impacted perceived foreign accent. In their study, NH listeners perceived low frequency words (i.e., infrequently occurring in a language) as more accented compared to high frequency words (i.e., commonly occurring in the language). Thus, real words appear to facilitate indexical processing, including talker discrimination, but lexical characteristics may also influence indexical processing.

Linguistic factors also impact response bias in talker discrimination in NH listeners. Previous research has reported that listeners are biased to perceive two words as produced by the same talker when these are lexically/semantically related (e.g., compounds) or are phonologically similar (e.g., rhymes) (Narayan *et al.*, 2017; Quinto *et al.*, 2020). El Adas and Levi (2022) found listeners were more likely to respond with “same talker” when listening to words compared to nonwords and for nonwords with high phonotactic probability compared to low phonotactic probability, in particular when talkers share similar voice characteristics (i.e., same gender). Finally, when presented with talker pairs in different languages, listeners are more likely to respond with “same talker” compared to “different talker” when the talkers speak the same language (Winters *et al.*, 2008).

### A. Talker discrimination in adult CI users

Less is known about the factors that impact talker discrimination in adult CI users. CIs restore a sense of hearing to adults with severe-to-profound hearing loss (HL). However, CI users rely on input signals that are heavily reduced in acoustic–phonetic detail compared to what is normally available to NH listeners, due to the limitations in information transmission of electric stimulation of the auditory nerve (see a review by Başkent *et al.*, 2016). As a group, CI users show a deficit in the perception of talker voice cues, such fundamental frequency (*F*0) and vocal tract length (VTL), in part due to the degraded spectro-temporal details transmitted by CIs and weak pitch perception (e.g., Fuller *et al.*, 2014; Gaudrain and Baskent, 2018; Meister *et al.*, 2016). As a consequence, compared to NH peers, CI users demonstrate a relative deficit in the perception of detailed talker-specific indexical information in speech (for a review, see Colby and Orena, 2022). Previous studies have shown that CI users, as a group, display difficulties in talker discrimination, in particular for the discrimination of same-gender talkers, who often share similarities in voice cues (Abdeltawwab *et al.*, 2016; Cleary *et al.*, 2005; Cleary and Pisoni, 2002; Hay-McCutcheon *et al.*, 2018; Li *et al.*, 2022; Massida *et al.*, 2011; Osberger *et al.*, 1991). In addition, CI users show less accurate or abnormal judgements for other sources of indexical variability, including a talker's gender (Fuller *et al.*, 2014; Massida *et al.*, 2013) or regional and foreign accent (Clopper and Pisoni, 2004; Hay-McCutcheon *et al.*, 2018; Tamati *et al.*, 2014; Tamati *et al.*, 2021). Finally, many CI users who use a bimodal configuration (i.e., CI + hearing aid in the non-implanted ear) may have access to low-frequency hearing that may facilitate the perception of talker-specific details (Başkent *et al.*, 2018; Hay-McCutcheon *et al.*, 2018). However, previous findings are mixed as to whether bimodal CI users achieve more accurate talker discrimination compared to other CI users, with some previous studies observing a bimodal benefit for talker discrimination accuracy (Hay-McCutcheon *et al.*, 2018) and other studies finding little evidence for significant differences among configurations (Abdeltawwab *et al.*, 2016; Cullington and Zeng, 2010, 2011). Additionally, bilateral CI users (i.e., with two CIs) may be able to utilize localization cues to facilitate talker discrimination in background noise or babble when spatially separated (e.g., Rana *et al.*, 2017), but these cues would not be available when the target talker and babble originate from the same direction. Taken together, these studies suggest that talker discrimination remains a significant real-world challenge for adult CI users, as a group, potentially regardless of configuration.

Although CI users display difficulties in talker discrimination, our understanding of the factors that contribute to their ability to discriminate talkers is still limited. CI users may take advantage of linguistic information in an effort to compensate for the relatively degraded talker information conveyed by the CI. Speech recognition can be effortful for CI users (Pals *et al.*, 2013; Winn *et al.*, 2015), and they rely on predictive coding and downstream cognitive processing resources to enhance the encoding and processing of the degraded speech input (e.g., Başkent *et al.*, 2016). CI users and NH listeners under CI simulation can make use of lexical knowledge to facilitate word recognition (e.g., Gianakas and Winn, 2019; Kaiser *et al.*, 2003; Sommers *et al.*, 1997; Tamati and Moberly, 2022). For indexical processing, one recent study showed that adult CI users and NH listeners under conditions of CI-simulated speech showed increased reliance on lexical-semantic cues and reduced reliance on prosodic cues to categorize emotions, relative to NH listeners under conditions of unprocessed speech (Richter and Chatterjee, 2021). Additionally, lexical content of words (forward vs time-reversed) has been shown to impact VTL perception in NH listeners under conditions of CI-simulated speech, with better perception in a just-noticeable differences (JND) task (i.e., smaller JNDs) for forward compared to reversed words (Koelewijn *et al.*, 2021). Together, this recent evidence suggests that CI users may make use of linguistic information to improve talker discrimination, but it is unclear how and when any effects may be observed.

The presence of noise or competing talkers may alter the effect of linguistic information on talker discrimination. The presence of background noise, such as MTB, represents one of the largest challenges for adult CI users, resulting in a significant detriment to speech recognition (e.g., Friesen *et al.*, 2001; Stickney *et al.*, 2004). Previous research suggests that CI users may be impaired in using voice cues to engage perceptual or linguistic mechanisms to segregate the target from the masking speech (e.g., El Boghdady *et al.*, 2019; Gaudrain *et al.*, 2007, 2008; Luo *et al.*, 2009). Similarly, MTB may also impact talker discrimination by further degrading voice and speech characteristics that can be used to discriminate talkers (Best *et al.*, 2018). Moreover, MTB may also impede the use of linguistic information in talker discrimination by masking the linguistic content of the utterance.

### B. The current study

The primary goals of the current study were to examine the impact of lexical content on talker discrimination in adult CI users, as a group, and to evaluate the extent to which the effect of lexical content varies by talker similarity and background noise. Experienced CI users with longer than one year of CI use completed an online AX talker discrimination task, in which they determined whether two different monosyllabic words were produced by the same or different talkers. To examine the effects of lexical content, word pairs were either lexically easy (high frequency, low neighborhood density) or lexically hard (low frequency, high neighborhood density) (Luce *et al.*, 1990; Luce and Pisoni, 1998). To evaluate whether the effect of lexical content depends on talker similarity, word pairs were produced either by the same talkers or different talkers with either the same gender (i.e., more similar voices) or mixed genders (i.e., more dissimilar voices). Finally, to examine the relative impact of lexical content by background noise, the task was completed in quiet and MTB.

Based on previous findings in NH listeners (El Adas and Levi, 2022; Narayan *et al.*, 2017; Quinto *et al.*, 2020), we expected that the lexical difficulty of the word pairs would impact talker discrimination in adult CI users and further that it should have a greater relative impact for talkers with similar voices (i.e., same-gender talker pairs). As for the direction of the effect, it was unclear whether accuracy would be higher for lexically easy or lexically hard words. On one hand, since hard words may be inaccurately recognized by adult CI users, it is possible that talker discrimination would be more accurate for lexically easy words than lexically hard words, consistent with previous findings showing better talker or voice cue discrimination for real words compared to nonwords and forward compared to time-reversed words (El Adas and Levi, 2022; Jebens *et al.*, 2022; Koelewijn *et al.*, 2021; Perrachione *et al.*, 2019). On the other hand, CI users may attend more to fine-grained phonetic details of lexically hard words, resulting in more accurate talker discrimination, consistent with some previous findings on accent perception (Levi *et al.*, 2007). Further, given recent findings suggesting that lexical and phonological factors may impact accuracy by altering response bias for phonologically similar words (El Adas and Levi, 2022), effects of lexical difficulty were expected to be observed on both accuracy and response bias. However, given the deleterious effect of MTB on speech recognition, we expected that adult CI users would be less able to access the lexical content of the target signals, resulting in a relatively weaker effect of lexical difficulty on talker discrimination in MTB. Finally, to consider one potential source of variability in talker discrimination among adult CI users, the impact of lexical difficulty on talker discrimination was explored across CI users with different device configurations (bilateral, bimodal, and unilateral).

## II. METHODS

### A. Participants

Twenty-four perilingually or postlingually deafened adult CI users (all with greater than one year of CI experience), participated in the current study. Participants (15 females/nine males) were between 41 and 83 yrs old, with a mean of 67 yrs [standard deviation (*SD)* = 9]. Duration of deafness prior to implantation, when available, was between 2 and 61 yrs, with a mean of 32 yrs (*SD* = 18). Two participants demonstrated a relatively shorter duration of deafness of less than 10 yrs, four participants demonstrated a duration of deafness between 10 and 20 yrs, four participants demonstrated a duration of deafness between 20 and 30 yrs, and ten participants demonstrated a duration of deafness of more than 30 yrs (four were unknown). All participants were implanted in adulthood, >18 yrs old. Seven participants were bilateral CI users, ten were bimodal CI users with a CI and contralateral hearing aid use, and seven were unilateral CI users with no contralateral hearing aid use. CI users were tested using their standard hearing devices (one CI, two CIs, or one CI with contralateral hearing aid, if typically worn) to represent their everyday listening conditions. Twenty-three of the participants had been recruited through the Ohio State University (OSU). They had previously participated in research studies at OSU and/or had previously indicated interest in participating in research studies. One participant was told about the study by an OSU participant and contacted the research team to participate. Participants provided informed written consent prior to participation and received $15 per hour for their time.

### B. Materials

Target words were monosyllabic English words produced by five female and five male talkers from the PB/MRT Word Multi-Talker Speech Database, based on the PB words (Egan, 1948) and MRT (House *et al.*, 1965), and selected as lexically easy and hard words (Kirk *et al.*, 1997). A total of 362 recordings (362 total words across talkers; 142 unique word items) were selected from the database, with 38 words for each talker. For each trial, participants were presented with a pair of two unique words, which were either both lexically easy or both lexically hard. Although some individual words were repeated (1–6 repetitions across listening conditions), these words were not repeated by the same talker within a trial or by the same talker within a single listening condition.

Words were presented in quiet or in co-located MTB. To generate the materials for the MTB listening condition, audio files containing the target words [equated to the same root mean square (RMS) amplitude level] were mixed with random samples from a 5-min stream of six-talker MTB, used in previous studies (Gilbert *et al.*, 2013; Tamati *et al.*, 2013). The MTB file was leveled in RMS amplitude to produce a signal-to-noise ratio (SNR) of +10 dB when mixed with the words. The MTB talkers were three male and three female speakers of Standard American English. The target word was preceded and followed by 500 ms of MTB alone. Finally, all materials (quiet and MTB) were leveled to the same RMS amplitude for consistency across listening conditions.

### C. Procedure

Testing was carried out remotely using the participants' own computers. Participants listened to stimuli presented over the computer speakers and did not stream directly to their processors. Before completing the talker discrimination task, participants completed a brief sound check in which they were asked to adjust the output volume. During the sound check, participants were first asked to adjust the volume to a comfortable level. Then, they were able to play two sentences as many times as necessary, while adjusting the output volume, until they were able to best understand the sentence. Participants were asked to keep the volume level the same throughout the remaining part of the testing session.

An AX same-different talker discrimination task was administered on the Gorilla Experiment Builder (www.gorilla.sc) online testing platform (Anwyl-Irvine *et al.*, 2020). The first block was completed in quiet, and the second block was completed in six-talker MTB at +10 dB SNR. Trials in both listening conditions consisted of presentation of two target words (in quiet or embedded in MTB), separated by 500 ms of silence. Prior to starting the task, participants were told to expect to hear two words that were either produced by the same talker or by two different talkers, and were instructed to indicate whether the words were produced by the same or different talkers by clicking on the corresponding button (“Same Talker” or “Different Talker”) on the screen. They were not told to expect any particular number of talkers or talker genders. The task was self-paced, and participants could continue to the next trial when ready.

Within each block, there were three talker pair conditions: same-talker (ST), different-talker, same-gender (DT-SG), and different-talker, mixed-gender (DT-MG), which varied by the similarity of the voices of the talker pairs. ST trials included target words produced by the same talker (i.e., same voice); DT-SG trials included target words produced by two different talkers who shared the same gender (i.e., more similar voices), and DT-MG trials included target words produced by a male and a female talker (i.e., more distinct voices).

The materials were designed to approximately balance ST trials and combined DT-SG and DT-MG trials (i.e., different-talker) across blocks. Each block consisted of 50 ST trials (100 ST total across blocks), 20 DT-SG trials (40 total across blocks), and 25 DT-MG trials (50 total across blocks), for a total of 95 trials in each block (190 total across blocks). Due to a coding error, nine items were not presented across both blocks (one ST, four DT-SG, four DT-MG; six in quiet, and three in MTB); since they were not presented, these items were excluded from analysis. Otherwise, all combinations of the ten talkers were presented within each task, as well as five repetitions of each ST pair. Additionally, items in each word pair were either both easy or both hard, as classified by the PB/MRT Word Multi-talker Speech Database, forming easy and hard lexical conditions, respectively.

## III. RESULTS

### A. Accuracy

**FIG. 1.**
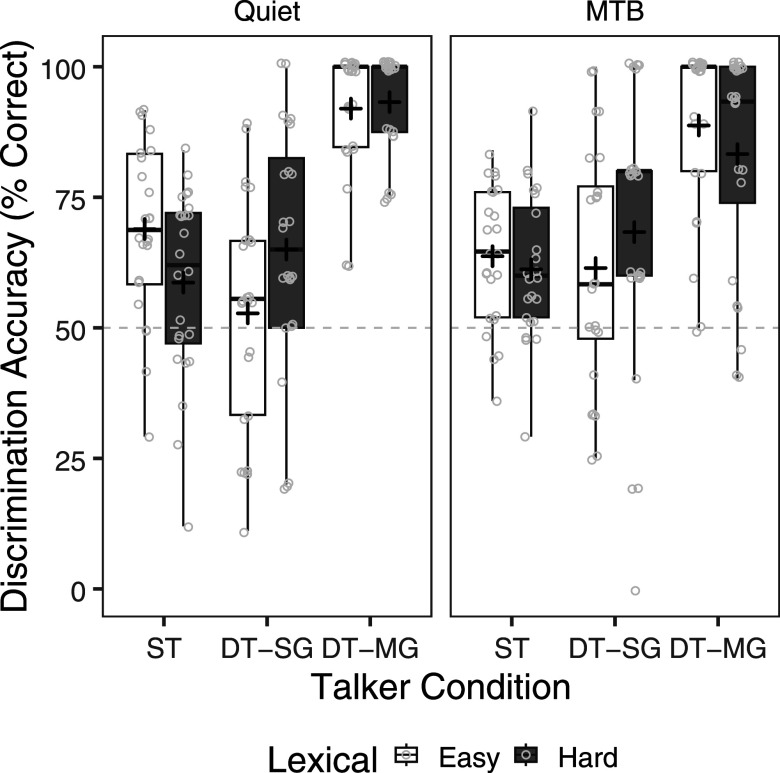
Talker discrimination accuracy (% correct) by talker condition (ST, DT-SG, DT-MG) and lexical condition (easy, hard) in quiet (left panel) and MTB (right panel). The boxes extend from the lower to the upper quartile (the interquartile range, IQ), the solid midline indicates the median, and the black plus sign indicates the mean. The whiskers indicate the highest and lowest values no greater than 1.5 times the IQ. Individual data points are represented by the light gray circles. Chance level of performance from random guessing (50%) is indicated by the light gray dashed line.

Figure 1 shows talker discrimination accuracy by talker condition and lexical condition in both quiet and MTB. A repeated measures analysis of variance (ANOVA) was carried out on discrimination accuracy with talker condition (ST, DT-SG, DT-MG), lexical condition (easy, hard), and listening condition (quiet, MTB) as within-subject factors. The analysis revealed a significant main effect of talker condition [*F*(2,46)= 24.22, *p* < 0.001], a significant two-way interaction of talker condition × listening condition [*F*(2,46) = 3.52, *p* = 0.038], and a significant two-way interaction of talker condition x lexical condition [*F*(2,46) = 15.82, *p* < 0.001]. The main effects of lexical condition [*F*(1,23) = 0.10, *p* = 0.752] and listening condition [*F*(1,23) = 0.18, *p* = 0.675] were not significant. The two-way interaction of lexical condition × listening condition [*F*(1,23) = 0.22, *p* = 0.643] and the three-way interaction of talker condition × lexical condition × listening condition [*F*(1,23) = 2.43, *p* = 0.099] also did not reach significance. Pairwise comparisons, with Bonferroni correction, showed that CI users were more accurate for DT-MG pairs compared to both DT-SG and ST pairs (all *p*'s < 0.001), but accuracy was not significantly different between DT-SG and ST pairs (*p* = 1.0). As can be seen in Fig. 1, participants performed relatively poorly in both the same-gender conditions, with 61.1% correct (SD = 18.8) for DT-SG pairs and 63.1% (SD =  3.0) for the ST pairs, close to performance if randomly guessing (50%). In contrast, participants were near ceiling performance on the DT-MG pairs with an average of 88.7% correct (SD = 13.7). For the two-way interaction of talker condition × listening condition, pairwise comparisons revealed that CI users were significantly more accurate in quiet than MTB for DT-MG pairs (*p* = 0.033), but not for DT-SG (*p* = 0.105) and ST pairs (*p* = 0.651). For the two-way interaction of talker condition × lexical condition, pairwise comparisons revealed that CI users were significantly more accurate for easy than hard words for DT-MG (*p* = 0.048) and ST pairs (*p* < 0.001), but were significantly more accurate for hard than easy words for DT-SG pairs (*p* = 0.004).

### B. Sensitivity and bias

To further examine the effect of lexical difficulty on talker discrimination, we evaluated sensitivity and bias for responses on same-gender (ST and DT-SG) trials. D-prime (d') sensitivity scores, which incorporate both identification rates (hits) and the false alarm rates, were calculated (Macmillan and Creelman, 2005). In the current study, hits refer to correct responses (i.e., different-talker responses) for different-talker pairs, and false alarms refer to incorrect responses (i.e., different-talker responses) for same-talker pairs. Beta response bias scores were also calculated to determine the participants' tendency to respond with same or different. The d' and beta scores were calculated using the R formulas from Pallier (2002).

**FIG. 2.**
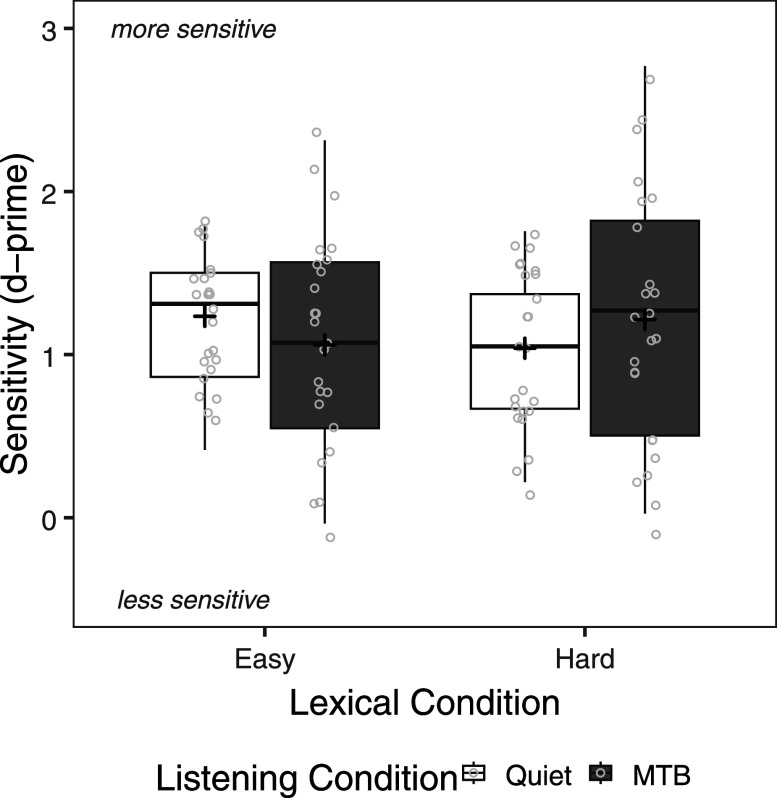
Sensitivity scores (d-prime) by lexical condition (easy, hard) and listening condition (quiet, MTB). The boxes extend from the lower to the upper quartile (the interquartile range, IQ), the solid midline indicates the median, and the black plus sign indicates the mean. The whiskers indicate the highest and lowest values no greater than 1.5 times the IQ. Individual data points are represented by the light gray circles.

Figure 2 shows sensitivity scores by lexical condition and listening condition. Note that higher scores indicate greater sensitivity. A repeated measures ANOVA was carried out on sensitivity scores with lexical condition (easy, hard) and listening condition (quiet, MTB) as within-subject factors. The analysis revealed a significant interaction of lexical condition × listening condition [*F*(1,23) = 5.98, *p* = 0.023]. Pairwise comparisons, with Bonferroni correction, showed that sensitivity was higher (i.e., higher scores) for easy compared to hard words in quiet (*p* = 0.030), but they were not significantly different in MTB (*p* = 0.177). The main effects of lexical condition [*F*(1,23) = 0, *p* = 1.00) and listening condition [*F*(1,23) = 0.09, *p* = 0.771] were not significant.

**FIG. 3.**
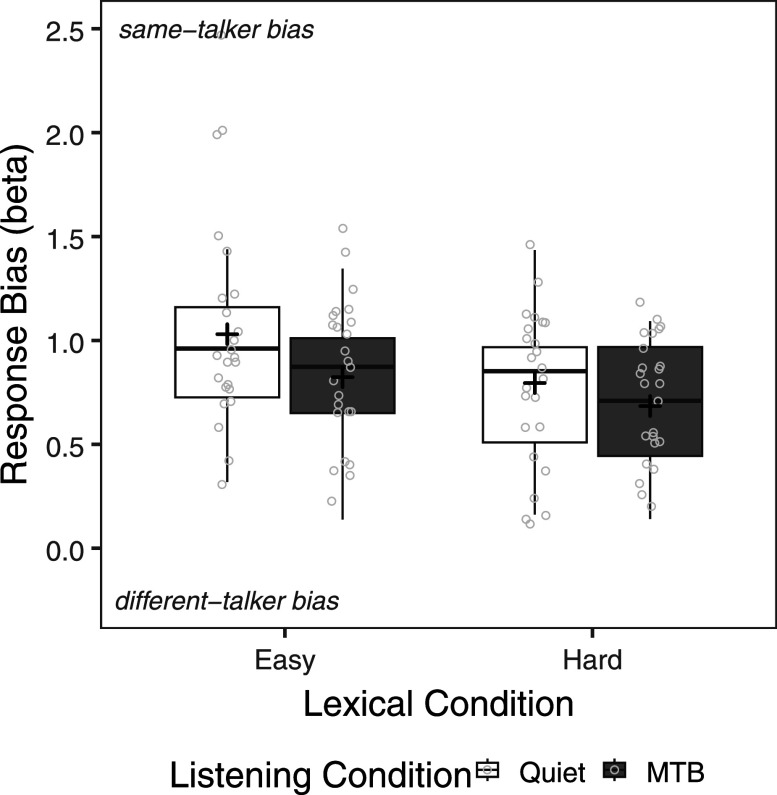
Response bias (beta) by lexical condition (easy, hard) and listening condition (quiet, MTB). The boxes extend from the lower to the upper quartile (the interquartile range, IQ), the solid midline indicates the median, and the black plus sign indicates the mean. The whiskers indicate the highest and lowest values no greater than 1.5 times the IQ. Individual data points are represented by the light gray circles.

Figure 3 shows response bias scores by lexical condition and listening condition. Note that a beta score of 1.0 represents an unbiased observer. Values above 1.0 represent a bias towards providing a same-talker response (i.e., lower hit rate and false alarm rate) and values below 1.0 represent a bias toward providing a different-talker response (i.e., higher hit rate and false alarm rate). A repeated measures ANOVA was also carried out on response bias scores with talker condition lexical condition (easy, hard) and listening condition (quiet, MTB) as within-subject factors. The analysis revealed a significant main effect of listening condition [*F*(1,23) = 6.25, *p* = 0.020] and a significant main effect of lexical condition [*F*(1,23) = 16.14, *p* < 0.001]. Pairwise comparisons showed that CI users demonstrated less bias overall (i.e., with scores closer to 1.0), having provided relatively fewer different-talker responses (or more same-talker responses), for easy compared to hard words (*p* = 0.020) and in quiet compared to MTB (*p* < 0.001). The interaction between lexical condition and noise condition was not significant [*F*(1,23) = 1.86, *p* = 0.185].

### C. Device configuration

To evaluate potential sources of differences among the CI users in the study, exploratory analyses were carried out on talker discrimination performance across the three device configurations (bilateral, bimodal, unilateral) used by the CI users in the current study. As stated above, seven participants were bilateral CI users, ten were bimodal CI users with a CI and contralateral hearing aid, and seven were unilateral CI users with no contralateral hearing aid. A series of one-way ANOVAs was carried out with listener group (bilateral, bimodal, unilateral) as the factor on sensitivity and response bias scores for lexically easy and lexically hard words in both quiet and MTB. Group differences on accuracy were not examined to reduce the total number of comparisons, and because performance in the DT-MG was close to ceiling. Results of the one-way ANOVAs showed that there was a significant effect of listener group on sensitivity for lexically hard words in MTB [*F*(2,21) = 5.2, *p* = 0.015]. The effect of listener group did not reach significance for any other condition. However, an inspection of average scores across listener groups, reported in Tables I and II, demonstrates an advantage for bilateral and bimodal CI users for sensitivity (d') compared to unilateral CI users, particularly for lexically hard words in both quiet and MTB (see Table I). In contrast, bilateral and bimodal CI users showed a bias towards different-talker responses relative to unilateral CI users.

**TABLE I.** Mean (SD) sensitivity (d') scores across listener groups (bilateral, bimodal, unilateral), and results of one-way ANOVAs with listener group as the factor. Significant effects of listener group are in bold.

Listening condition	Lexical condition	Bilateral (CI + CI)	Bimodal (CI + hearing aid)	Unilateral (CI only)	Listener group effect (one-way ANOVA)
Quiet	Easy	1.31 (0.27)	1.33 (0.37)	1.03 (0.50)	*F*(2,21) = 1.4, *p* = 0.279
	Hard	1.20 (0.49)	1.17 (0.42)	0.69 (0.32)	*F*(2,21) = 3.4, *p* = 0.052
MTB	Easy	1.11 (0.90)	1.2 (0.48)	0.75 (0.57)	*F*(2,21) = 1.2, *p* = 0.321
	Hard	1.30 (0.79)	1.61 (0.72)	0.56 (0.38)	***F*(2,21) = 5.2, *p* = 0.015**

**TABLE II.** Mean (SD) response bias (beta) scores across listener groups (bilateral, bimodal, unilateral), and results of one-way ANOVAs with listener group as the factor. Significant effects of listener group are in bold.

Listening condition	Lexical condition	Bilateral (CI + CI)	Bimodal (CI + hearing aid)	Unilateral (CI only)	Listener group effect (one-way ANOVA)
		0.97 (0.52)	0.88 (0.53)	1.30 (0.45)	*F*(2,21) = 1.5, *p* = 0.242
		0.74 (0.35)	0.66 (0.38)	1.05 (0.14)	*F*(2,21) = 3.2, *p* = 0.062
		0.73 (0.28)	0.74 (0.30)	1.03 (0.19)	*F*(2,21) = 2.9, *p* = 0.080
		0.62 (0.28)	0.58 (0.33)	0.90 (0.17)	*F*(2,21) = 3.0, *p* = 0.073

## IV. DISCUSSION

The current study examined the effect of lexical difficulty on talker discrimination in adult CI users, as a group, and the extent to which the effect of lexical content varies by talker similarity and the presence of MTB. First, we observed that lexical difficulty impacted talker discrimination accuracy in both quiet and MTB, with the strongest effects observed for same-gender talker pairs. More specifically, CI users were more accurate for easy words compared to hard words when produced by same-talker pairs or different-talker, mixed-gender pairs. However, they were also more accurate for hard words compared to easy words for different-talker, same-gender pairs. Second, the effect of lexical difficulty on same-gender talker discrimination was further analyzed by evaluating sensitivity and bias scores for same-gender pairs only. Lexical difficulty impacted sensitivity scores, but its effect depended on the presence of noise. CI users were more sensitive to talker differences for easy words compared to hard words in quiet but not in MTB. Additionally, there was an effect of lexical difficulty on response bias, such that CI users were less biased to give different-talker responses for lexically easy compared to lexically hard words. As such, this finding for response bias is consistent with the observed effect of lexical difficulty on same-gender talker discrimination accuracy. A tendency to give relatively fewer different-talker responses (or more same-talker responses) for lexically easy words would result in more accurate responses for same-talker pairs and less accurate responses for different-talker, same-gender pairs, compared to lexically hard words. Interestingly, the effect of lexical difficulty on same-gender talker discrimination did not appear to depend on the presence of MTB. Thus, there was an effect of lexical difficulty on response bias that depended on talker similarity.

The effect of lexical difficulty on talker discrimination, in particular for same-gender pairs, is partially consistent with our hypothesis. We expected that adult CI users would utilize linguistic information as a compensatory strategy in talker discrimination, given that they must rely on degraded talker-specific cues. Previous studies have demonstrated that CI users make use of linguistic content (e.g., semantic or lexical content) of an utterance to facilitate spoken word or sentence recognition (Moberly and Reed, 2019; O'Neill *et al.*, 2021; Taitelbaum-Swead *et al.*, 2022; Tamati and Moberly, 2022). Although fewer studies have examined the effect of linguistic content on indexical processing, Richter and Chatterjee (2021) found CI users displayed increased reliance on lexical–semantic content and reduced reliance on prosodic cues in emotion identification, compared with NH listeners. Similar to talker discrimination, acoustic–phonetic prosodic details cueing different emotions are also poorly conveyed by the CI, resulting in relatively challenging emotion categorization for adult CI users. Thus, taken together, adult CI users may rely more heavily on the linguistic content of an utterance when processing degraded talker information.

Interestingly, lexical difficulty appeared to have a relatively greater impact on same-gender talker discrimination. Previous studies have consistently demonstrated that adult CI users demonstrate relatively good discrimination accuracy for mixed-gender talker pairs, whose voice and speech characteristics are more disparate compared to same-gender talker pairs (Abdeltawwab *et al.*, 2016; Cleary *et al.*, 2005; Cleary and Pisoni, 2002; Hay-McCutcheon *et al.*, 2018; Li *et al.*, 2022; Massida *et al.*, 2011; Osberger *et al.*, 1991). In the current study, CI users were more accurate for mixed-gender talker pairs compared to same-gender talker pairs (See Fig. 1). Although we observed that accuracy was also slightly higher for easy compared to hard words for mixed-gender pairs, ceiling effects may have limited the effect of lexical difficulty on accuracy in that condition. In other challenging listening conditions, lexical difficulty may play a stronger role in mixed-gender talker discrimination. For the current study, our finding suggests that lexical content has a greater relative impact for talkers with similar voices (i.e., same-gender talker pairs) in quiet and MTB, when discrimination is more challenging.

In the current study, we observed that talker discrimination accuracy was higher for easy compared to hard words for same-talker pairs but was higher for hard compared to easy words for different-talker, same-gender trials. Examining sensitivity and response bias, the results for same-gender talker discrimination accuracy may be explained both by greater sensitivity to easy compared to hard words and by response bias. We observed an effect of lexical difficulty on sensitivity for same-gender pairs, specifically in quiet, suggesting that overall CI users may be more sensitive to talker differences for easy words, particularly in more favorable conditions. This is also consistent with talker discrimination accuracy for mixed-gender talkers, where accuracy was higher for easy compared to hard words. However, the seemingly contrasting effects of lexical difficulty on same-talker and different-talker, same-gender pairs can likely be explained by response bias. CI users demonstrated less response bias for lexically easy words compared to lexically hard words. Interestingly, CI users also demonstrated relatively less bias overall in quiet than in MTB. Thus, when words were easier to process and likely more discriminable for adult CI users, they perceived the voices as being less different. Without access to reliable talker-specific information for same-gender talker discrimination, CI users made use of the lexical information available to them to make talker judgements. Similarly, El Adas and Levi (2022) found that NH participants were more likely to give same-talker responses for real words compared to nonwords, when items were blocked; when real words and nonwords were randomly presented, while there was no overall effect of word status (real words vs nonwords), participants were more likely to provide same-talker responses for nonwords that were less similar to real words (with high phonotactic probability). Quinto and colleagues (2020) also reported a lexical effect on response bias. Listeners were more likely to perceive talkers as the same when listening to compounds, compared to unrelated word pairs.

Taken together, our results also suggest an overall processing benefit for easy compared to hard words, both for same-gender pairs and mixed-gender pairs. The finding that lexically easy words may improve sensitivity to talker details, in certain conditions, is seemingly consistent with previous results demonstrating that certain lexical and/or phonological properties facilitate talker discrimination. El Adas and Levi (2022) additionally found a faciliatory effect for word status (real words vs nonword) and high phonological probability on talker discrimination, observing relatively shorter response times for real words, overall, and real words with high phonotactic probability compared to real words with low phonotactic probability. However, this effect of word status and phonotactic probability only emerged when items were presented in blocks by word status (meaning words and nonwords separately), and not when they were randomized. Further, when accuracy is relatively high, such as for mixed-gender talker discrimination, lexical effects may be better observed on response times, which were not investigated in the current study. Methodological differences impede our ability to directly relate our findings to these earlier studies to investigate the extent to which NH listeners and CI users make use of similar or different strategies in talker discrimination. Future studies should more directly investigate the effects of lexical and phonological content on talker discrimination in adult CI users and their NH peers.

Finally, we also sought to determine whether the effect of lexical difficulty depended on the presence of MTB. We hypothesized that lexical difficulty would show a relatively weaker effect on talker discrimination in MTB. Our findings were partially consistent with this hypothesis. When examining sensitivity scores, we observed that sensitivity was higher for easy than hard words in quiet but not in MTB. Thus, MTB appears to have affected CI users' ability to use lexical content to facilitate talker discrimination. Competing speech represents a significant challenge to spoken word recognition in adult CI users, due to limitations in the use of voice cues for separating target from masking speech (e.g., El Boghdady *et al.*, 2018; Gaudrain *et al.*, 2007, 2008; Luo *et al.*, 2009). The results of the current study suggest that by obscuring the linguistic content of the utterance, MTB may also limit its use in talker discrimination. As such, limitations in voice cue perception may impact talker discrimination in MTB both directly, by limiting the ability to detect differences in voice cues across talkers, and indirectly, by reducing the ability to make use of linguistic information as a compensatory strategy. Interestingly, however, overall talker discrimination performance (accuracy or sensitivity) did not display an overall decline in MTB, contrary to previous findings in hearing-impaired listeners (Best *et al.*, 2018). One possible account is that the voice cue(s) relied upon to discriminate talkers in adult CI users may be relatively impervious to masking by MTB. Adult CI users have been shown to rely relatively more heavily on *F*0 compared to VTL in voice gender perception, compared to NH listeners (Fuller *et al.*, 2014). CI users may also rely more heavily on *F*0 to make talker discrimination judgements, as compared to VTL or other speech cues that may be more affected by masking (Winn *et al.*, 2013). However, the effects of MTB on overall performance should be interpreted with caution. It is important to note that accuracy was relatively poor for same-gender talker pairs and also quite good (close to ceiling) for mixed-gender talker pairs in both quiet and MTB. Therefore, it is also possible that the effects of MTB on talker discrimination may have been obscured due to floor and ceiling effects. More research should be carried on talker and voice cue perception in real-world challenging conditions to better understand their overall effects on performance as well as their effects on compensatory strategies in indexical processing.

Finally, exploratory analyses were conducted to evaluate the potential effect of device configuration on talker discrimination. Although a significant effect of listener group (bilateral, bimodal, unilateral) emerged only for sensitivity (d') scores for lexically hard words in MTB, a comparison of average scores across conditions (see Table I) suggests that bilateral and bimodal CI users demonstrated greater sensitivity for same-gender talker discrimination compared to unilateral CI users. Interestingly, the group differences appeared to be greater for lexically hard word pairs compared to lexically easy word pairs. Additionally, bilateral and bimodal CI users appeared to be more biased to give different-talker responses than unilateral CI users. These exploratory analyses suggest that both bilateral and bimodal CI users utilize linguistic information in talker discrimination to a greater extent than unilateral CI users, potentially due to greater access to the linguistic content of the utterance. Bilateral and bimodal CI users tend to have better speech recognition outcomes than unilateral CI users (e.g., Blamey *et al.*, 2015; Sturm *et al.*, 2020). However, the results of the exploratory analyses should be interpreted with caution given the small number of participants per device configuration, and given that this study was not specifically designed to examine how lexical difficulty and noise may interact with device configuration.

Broadly, the results of the current study demonstrate that the linguistic content impacts talker discrimination in adult CI users, as a group. These findings suggest that CI users may be able to make use of linguistic information to facilitate indexical processing to compensate for the relatively degraded indexical information conveyed by the CI. However, we have noted some points of our study that can be improved in future research. First, although outside the scope of the current study, we observed substantial individual differences in performance that may be related to factors beyond device configuration. Adult CI users show a large age range and diverse language background and experiences, duration of severe HL prior to implantation, duration of CI use, and residual hearing, among other factors (e.g., Blamey *et al.*, 2015; Heydebrand *et al.*, 2007). In particular, the adult CI users in the current study as a group demonstrated a long duration of deafness prior to receiving a CI (M = 32 yrs; *SD* = 18), but also showed substantial variability with 2–61 yrs of deafness prior to receiving a CI. As observed in previous studies (e.g., Blamey *et al.*, 2015), longer duration of deafness is associated with relatively poorer speech recognition outcomes in adult CI users. However, the contribution of duration of deafness to indexical processing, and specifically talker discrimination, has not been investigated. Individual factors could be better controlled or directly investigated in future studies. For example, future research examining performance over time in a longitudinal design among diverse CI populations including postlingually, perilingually, and prelingually deafened adult CI users is needed to better understand the individual factors that impact talker discrimination in adult CI users.

## V. CONCLUSION

The current study examined the impact of lexical content on talker discrimination in adult CI users, and the extent to which the effect of lexical content varies by talker similarity and background noise. Adult CI users completed a remote AX talker discrimination task with pairs of lexically easy (high frequency, low neighborhood density) or lexically hard (low frequency, high neighborhood density) words. Word pairs were produced either by the ST or DT-SG or (DT-MG) and were presented in quiet and MTB. Results demonstrated that lexical difficulty impacted talker discrimination, particularly for same-gender talker pairs. For lexically easy words, CI users were more accurate for same-talker pairs and less accurate for different-talker, same-gender pairs. However, there was an effect of lexical difficulty on response bias, such that CI users were less biased for lexically easy words. An overall processing benefit for easy compared to hard words was also observed, when examining sensitivity for same-gender talker discrimination and accuracy for mixed-gender talker discrimination. Finally, while no overall performance differences were observed between talker discrimination in quiet and MTB, the presence of MTB appeared to impede the use of lexical content in talker discrimination to some extent. These results suggest that CI users make use of lexical content for discriminating talker pairs with similar voices (i.e., same-gender talker pairs), and are more sensitive to talker-specific details in talker discrimination when lexical processing is facilitated by lexically easy words, particularly in quiet. Overall, these findings provide evidence for the contribution of linguistic information to the processing of degraded talker information by adult CI users.

## Data Availability

The data that support the findings of the study are available from the corresponding author upon reasonable request.
